# Cu(ii) binding to an antimicrobial shrimp peptide – a small step for structural chemistry, a big leap for medicinal applications†

**DOI:** 10.1039/d4sc05222f

**Published:** 2025-01-23

**Authors:** Adriana Miller, Agnieszka Matera-Witkiewicz, Aleksandra Mikołajczyk-Tarnawa, Arian Kola, Magdalena Wiloch, Martin Jonsson-Niedziolka, Robert Wieczorek, Joanna Wątły, Daniela Valensin, Magdalena Rowińska-Żyrek

**Affiliations:** a Faculty of Chemistry, University of Wrocław ul. F. Joliot-Curie 14 50-383 Wrocław Poland magdalena.rowinska-zyrek@uwr.edu.pl; b Screening of Biological Activity Assays and Collection of Biological Material Laboratory, Wrocław Medical University Biobank, Faculty of Pharmacy, Wrocław Medical University ul. Borowska 211A 50-556 Wrocław Poland; c Department of Biotechnology, Chemistry and Pharmacy, University of Siena Via Aldo Moro 2 53100 Siena Italy; d Institute of Physical Chemistry, Polish Academy of Sciences ul. Marcina Kasprzaka 44/52 01-224 Warszawa Poland; e Consorzio Interuniversitario Risonanze Magnetiche di Metalloproteine (CIRMMP) Via L. Sacconi 6 50019 Sesto Fiorentino Italy

## Abstract

PvHCt, a 23-amino acid long, histidine-rich peptide derived from shrimp, becomes strongly antimicrobial upon Cu(ii) ion binding. We describe Zn(ii) and Cu(ii) complexes of this peptide, aiming to understand how metal binding and structure correlates to biological activity. Using NMR, UV-vis, CD and FTIR spectroscopies, along with cyclic voltammetry, potentiometry, and DFT calculations, we demonstrate that Cu(ii) binds to the central and C-terminal regions of the peptide, inducing significant structural changes. These include a pronounced bend in the peptide backbone, increased α-helical content, and the production of reactive oxygen species, all of which contribute to the remarkable antimicrobial potency of PvHCt. In contrast, Zn(ii) binds to the C-terminal region with minimal impact on the peptide's overall structure, failing to enhance its antimicrobial activity.

## Abstract 2

PvHCt, a 23-aa AMP from shrimp, becomes strongly antimicrobial when Cu(ii) binds to its central and C-terminal regions, inducing a pronounced bend in the peptide backbone, increased α-helical content, and production of reactive oxygen species.

## Graphical abstract

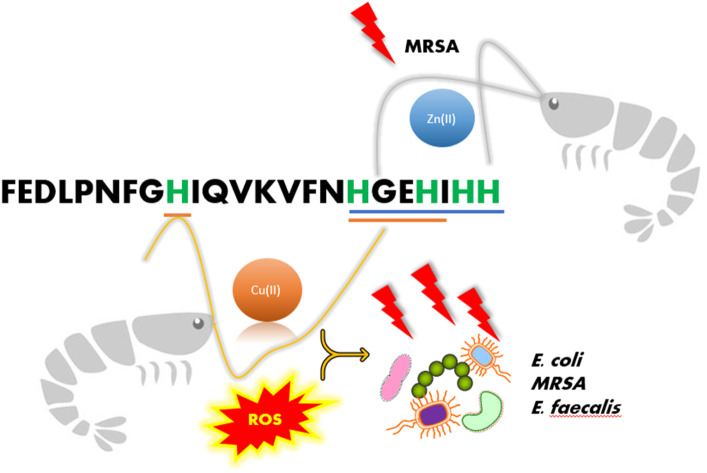

## Introduction

Antimicrobial peptides (AMPs) are a potential treasure trove for the design of novel, effective therapeutics aimed to fight antibiotic-resistant pathogens, a major challenge in current clinical treatment. AMPs are present in numerous organisms from various habitats, and the marine environment seems to be a generous source of such bioactive molecules – divergent AMPs are found in oceanic invertebrates, fish, amphibians, reptiles, and mammals. In the literature, this environment is even considered to be the ‘food and medicine bank of the new millennium’.^1^ Shrimp antimicrobial peptides, the major focus of this work, have a ‘double importance’ – first, they are crucial for the well-being of shrimps themselves and second, they may be a potential source for antimicrobial human therapeutics.

As the fastest-growing food production system, aquaculture is becoming increasingly significant in the global food supply. Due to the high content of bioavailable nutrients and high accessibility in low- and medium-income regions, aquatic food is a precious weapon in the fight with malnutrition.^2,3^ In the last 30 years, aquaculture production has increased from 13.1 million (1990) to 82.1 million metric tons (2018),^2,4^ with shrimps accounting for a significant percentage of these numbers.

Even despite introducing many strategies preventing the spreading of infection,^4–9^ shrimp farms are bothered by a variety of pathogens, like *Enterocytozoon hepatopenaei*, *Vibrio parahaemolyticus* or White Spot Syndrome Virus, which cause the most common shrimp diseases and also significant economic losses, counted in billions of dollars per year.^4,10^

Since crustaceans did not develop an adaptive immune system, their innate immune response is crucial for fighting pathogens.^11^ Their defensive mechanisms are mainly divided into cellular and humoral immune responses. When the first includes cell-dependent processes, such as phagocytosis, the latter focuses on producing bioactive molecules like, for example, antimicrobial peptides.^11,12^

Penaeid shrimps produce antimicrobial peptides, most of which are gene-encoded. Among this group, penaedins, crustins and stylicins can be found.^11^ Moreover, some of the antimicrobial peptides can be the result of proteolytic cleavage of larger proteins. This kind of synthesis is observed for PvHCt peptide, which is obtained from the C-terminal fragment of hemocyanin from shrimp *Litopenaeus vannamei*.^13^ Hemocyanins are copper-binding proteins and the most abundant protein component in crustaceans' hemolymph (around 95%).^14^ Their main function is the transport of oxygen, but they can also be involved in osmoregulation, the molt cycle, formation of the exoskeleton and immune response.^15–17^

**Fig. 1:**
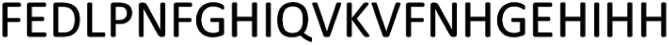
Amino acid sequence of PvHCt peptide (PDB ID: 2N1C)^18^

PvHCt is a 23-amino acid long, histidine-rich peptide (Fig. 1), which adapts an α-helical structure in a membrane-mimicking environment.^13^ To date, its antifungal activity against *Fusarium oxysporum* (a fungal plant pathogen) was observed, without any activity against Gram-positive or Gram-negative bacteria.^13,19^ Interestingly, in the case of infection with the White Spot Syndrome Virus, hemocyanin from *L*. *vannamei* is cleaved to several antimicrobial peptides. One of them is a C-terminal, 79-amino acid fragment named LvHcL48 (ref. 20) which inhibits the transcription and proliferation of the White Spot Syndrome Virus. Moreover, the last 23 residues from the LvHcL48 are in approximately 96% agreement with the PvHCt sequence, differing only with Q11-R substitution.^20^

Since the coordination of metal ions can, in numerous cases, improve the biological activity of antimicrobial peptides,^21^ (also aquatic ones)^22,23^ we were intrigued with the possible impact of Zn(ii) and Cu(ii) on the PvHCt's activity against not only shrimp, but also human pathogens. Knowing that hemocyanins are able to bind essential metal ions,^14^ we decided to check if and how the peptide hydrolyzed from the C-terminal hemocyanin fragment from *L. vannamei* coordinates Zn(ii) and Cu(ii) ions, which are highly abundant in penaeid tissues.^24^ The possibility of Zn(ii) and Cu(ii) coordination is also strongly suggested by the presence of specific zinc and copper binding motifs (pneumococcal His-triad: -HxxHxH and bis-His motif HH).^25–29^

## Results

### Ligand deprotonation

For the PvHCt peptide (FEDLPNFGHIQVKVFNHGEHIHH), nine deprotonation constants are determined (Table 1). The first two constants are assigned to the deprotonation of glutamic acid side chains (p*K*_a_ equal 4.04 and 5.02). The next five constants correspond to the deprotonation of five imidazole groups from histidine side chains (p*K*_a_ equal 5.56, 6.70, 7.79, 7.89 and 8.47). The next constant (for the H_2_L form) indicates the deprotonation of the N-terminal amine group (p*K*_a_ = 8.80), and the last value comes from the deprotonation of lysine residue, with a p*K*_a_ value equal 9.72.

**Table 1:** Protonation and stability constants for PvHCt peptide (FEDLPNFGHIQVKVFNHGEHIHH) and its Zn(ii) and Cu(ii) complexes. Titrations were carried out over the pH range 2–11 at *T* = 298 K in an aqueous solution with 4 mM HClO_4_ and 40 mM SDS as ionic strength. The peptide concentration was 0.5 mM and the Zn(ii)- and Cu(ii)-to-peptide ratio was 1 : 1. HYPERQUAD 2006 (ref. 30) was used to determine the stability constants. Standard deviations are shown in brackets

Species	Log β	p*K*_a_	
**PvHCt FEDLPNFGHIQVKVFNHGEHIHH**			
H_9_L	63.99 (7)	4.04	–COOH (Glu)
H_8_L	59.95 (5)	5.02	–COOH (Glu)
H_7_L	54.93 (5)	5.56	–N_im_ (His)
H_6_L	49.37 (4)	6.70	–N_im_ (His)
H_5_L	42.67 (3)	7.79	–N_im_ (His)
H_4_L	34.88 (3)	7.89	–N_im_ (His)
H_3_L	26.99 (3)	8.47	–N_im_ (His)
H_2_L	18.52 (2)	8.80	–NH_2_
HL	9.72 (2)	9.72	–*ε*NH_2_ (Lys)

**Zn(II) complexes**			
ZnH_5_L	46.46 (6)		
ZnH_4_L	—		
ZnH_3_L	33.22 (4)		
ZnH_2_L	25.90 (5)	7.31	–N_im_ (His)
ZnHL	18.34 (4)	7.56	(H_2_O)
ZnL	9.89 (6)	8.45	–NH_2_
ZnH_-1_L	—		
ZnH_-2_L	−9.97 (6)		

**Cu(II) complexes**			
CuH_6_L	52.40 (6)		
CuH_5_L	46.39 (5)	6.02	–N^−^
CuH_4_L	39.84 (7)	6.55	–N_im_ (His) non binding
CuH_3_L	33.27 (4)	6.57	–N_im_ (His)
CuH_2_L	26.06 (3)	7.21	–N_im_ (His)
CuHL	18.62 (2)	7.44	–N_im_ (His)
CuL	10.11 (3)	8.51	–NH_2_
CuH_-1_L	0.44 (3)	9.67	–N^-^
CuH_-2_L	−9.86 (3)	10.29	–N^-^
CuH_-3_L	−20.31 (3)	10.46	–*ε*NH_2_ (Lys)

### Zn(ii) complexes

According to the mass spectrometry results, the PvHCt peptide forms complexes with Zn(ii) only in a mononuclear form, with a metal:ligand ratio 1 : 1 (Fig. S1A and B†). Zinc(ii) starts to coordinate at pH around 5.5, forming a ZnH_5_L complex with a log *β* value 46.46 (Table 1 and Fig. S2A†).

With the increase of pH, ZnH_3_L species are observed with log *β* of 33.22. This form dominates at pH around 7. At this point, all acidic residues are deprotonated and Zn(ii) is probably coordinated by two imidazole nitrogens from histidine side chains.

**Fig. 2:**
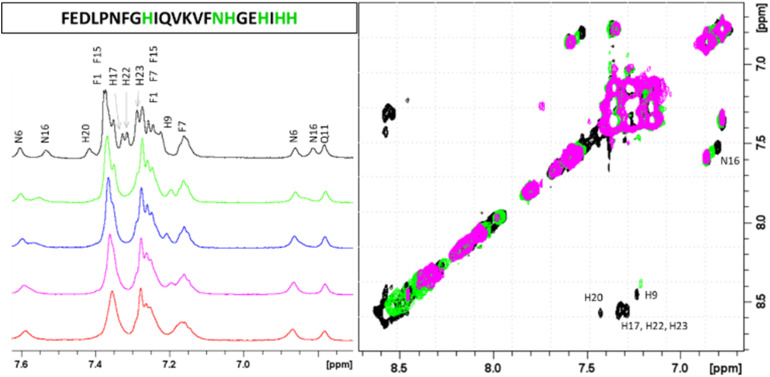
Aromatic regions of 1D ^1^H and 2D ^1^H-^1^H TOCSY spectra of PvHCt 0.5 mM, SDS-d_26_ 40 mM, pH 7.4 T 298 K in absence (black) and in presence of 0.2 (green), 0.4 (blue), 0.6 (magenta) and 0.8 (red) zinc(ii) equivalents. The most affected residues of the peptide sequence are shown in green.

The next form, ZnH_2_L is the most abundant at pH around 7.4. Comparison of the p*K*_a_ values for the free ligand and the Zn(ii) complex (8.47 and 7.31 respectively) suggests the coordination of the third imidazole nitrogen, what is also confirmed by NMR. Both 1D and ^1^H-^1^H TOCSY spectra registered around pH 7.4 show that addition of Zn(ii) to PvHCt solutions caused chemical shift variations and broadening of selected NMR resonances as usually detected for other zinc(ii)-peptide associations.^31^ The metal effects were immediately evident upon the addition of 0.2 metal equivalents, leading to the complete disappearance of aromatic protons belonging to H17, H20, H22 and H23 and chemical shift variations and much less pronounced broadening on H*δ* signals of H9 and N16 (Fig. 2).

**Fig. 3:**
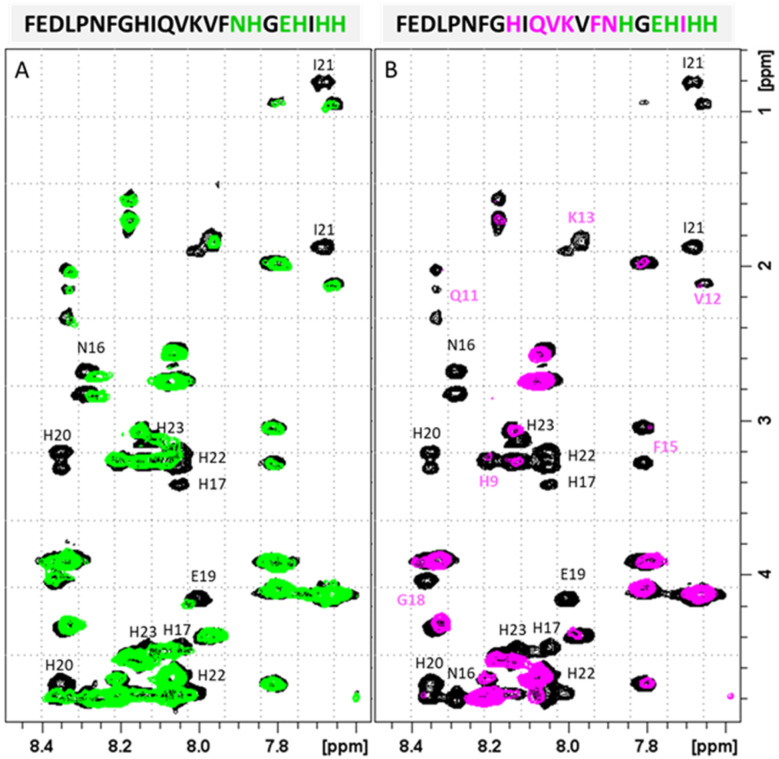
Finger-print regions of 2D ^1^H-^1^H TOCSY spectra of PvHCt 0.5 mM, SDS-*d*_26_ 40 mM, pH 7.4, T 298 K in absence (black) and in presence of (A) 0.2 Zn(ii) eqs (green) and (B) 0.6 Zn(ii) eqs (magenta). The most affected residues of the peptide sequence are shown in green and magenta for 0.2 and 0.6 eqs additions, respectively.

**Fig. 4:**
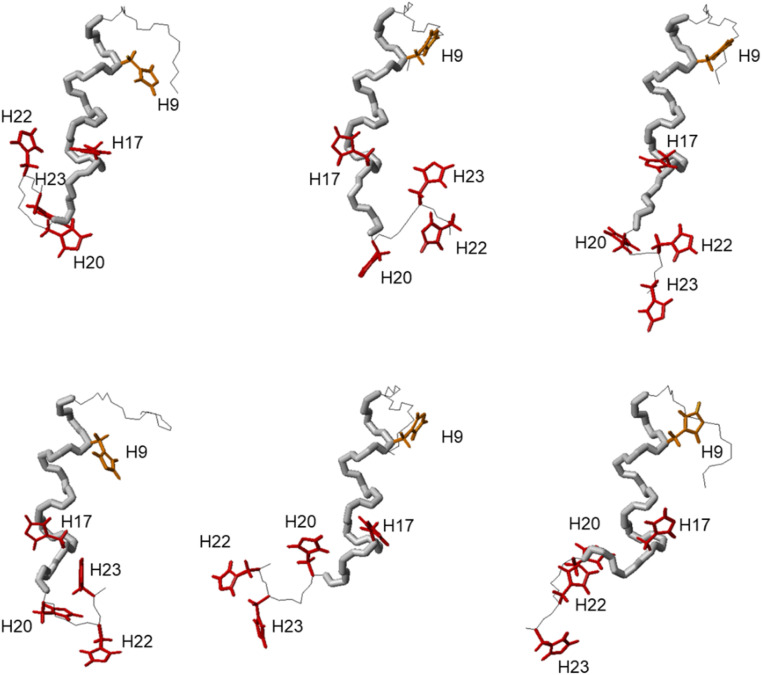
Structures n° 1, 4, 8, 10, 11 and 12 extracted from PDB ID 2N1C.^18^ The different orientation of H9, H17, H20, H22 and H23 are shown for all the selected structures.

**Fig. 5:**
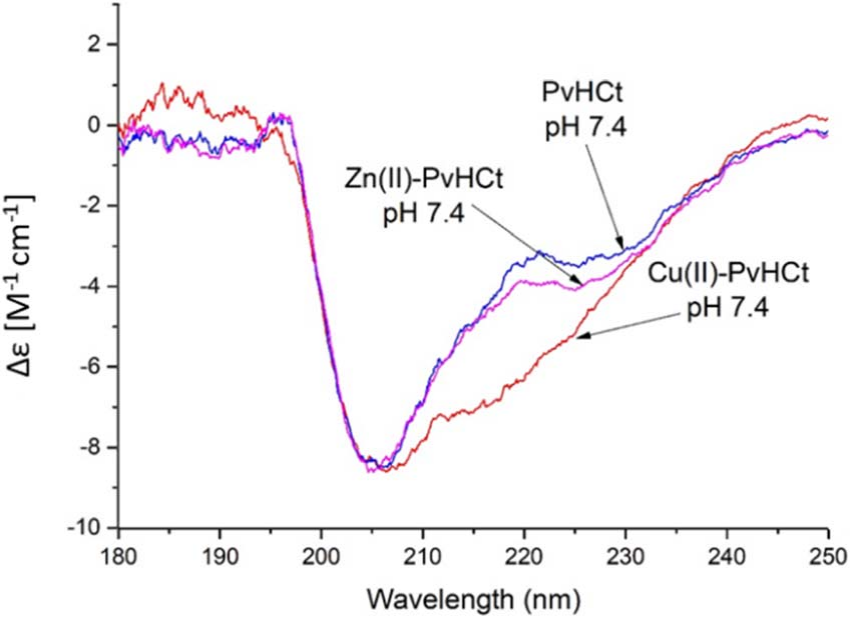
Analysis of secondary structure at pH 7.4 for PvHCt peptide (blue) and its Zn(ii) (magenta) and Cu(ii) (red) complexes. Addition of Cu(ii) ion causes a significant increase of percentage of α-helical structure. *T* = 298 K, *I* = 40 mM SDS, [*L*] = 0.5 mM; metal-to-ligand ratio M(II) : *L* = 0.9 : 1.

Similarly, the comparison between the finger-print regions of ^1^H-^1^H TOCSY spectra recorded in absence and in presence of 0.2 Zn(ii) eqs points out the vanishing of the cross-peaks belonging to H17, E19, H20, I21, H22 and H23, strongly suggesting the involvement of the C-terminal part in the Zn(ii) coordination sphere (Fig. 3A), but not giving a direct answer about the number of bound imidazoles. Increasing zinc(ii) concentrations up to 0.6 eqs leads to the further loss of the signals corresponding to H9, Q11, V12, K13 and F15 (Fig. 3B), probably indicating the subsequent occupancy of the metal to a second binding site located around H9. In fact, the simultaneous zinc coordination to H9 and the C-terminal H17, H20 and H22 was ruled out because of the helical structuring of the peptide which hampers the folding of the central amino acids around the zinc ion without inducing additional structural changes – according to the literature,^13,18^ in the free PvHCt, the C-terminal His residues experience flexible and different orientations, with multiple degrees of freedom to achieve the necessary coordination sphere (Fig. 4). In contrast, the region between residues 9–17 adopts an α-helical conformation, which restricts the movements of H9 sidechain. To engage imidazole nitrogens from H9 and C-terminal His residues in coordination at the same time, a structural change would be needed. According to the CD spectra (Fig. 5), as the structure does not change after the addition of Zn(ii), therefore, the simultaneous binding of H9 and the C-terminal imidazoles from the unstructured C-terminus can be ruled out, strongly indicating that the coordination of zinc(ii) occurs only *via* the C-terminal His residues (H17, H20, H22 and H23).

On the other hand, data obtained from competition diagram excludes the 4N mode of coordination in Zn(ii)-PvHCt complex.

This diagram (Fig. S3†), based on the calculated stability constants, presents a hypothetical situation when equimolar amounts of reagents are mixed and enables to compare the stability of examined complexes. A comparison of the stability of complexes involving respectively 3 and 4 imidazole nitrogens in Zn(ii) coordination shows that PvHCt has lower affinity for Zn(ii) than the 3N-coordinating clavanin D, strongly suggesting the formation of the so-called polymorphic states (with at least 2N type of Zn(ii) binding) – the metal coordinates to different sets of binding sites, forming multiple complex forms, which coexist in solution in equilibrium. In case of the Zn(ii)-PvHCt complex, at least two 2N-type species are most likely present in the solution, each involving a different set of three C-terminal imidazoles in Zn(ii) binding.

DFT calculations clarified that ‘at least two’ means precisely ‘three’. All three complexes display 2N type of connections supported by metal–oxygen interactions form carbonyl groups. In the first type of complex (complex A), Zn(ii) is bound to His20 and His23, in complex B – to His22 and His23, and in complex C – to His17 and His22, as presented in Table 2 and Fig. S4.†

**Table 2:** Metal – ligand distances in angstroms of the *A*, *B*, *C* Zn(ii)- FEDLPNFGHIQVKVFNHGEHIHH complexes

	*A*	*B*	*C*
H23 (imidazole)	1.938	1.921	
H22 (imidazole)		2.057	1.904
H20 (imidazole)	2.037		
H17 (imidazole)			2.032
H23 (C=O)	2.089	2.096	
F15 (C=O)	2.089	1.984	
V14 (C=O)			1.957

Loss of the next proton results in the formation of the ZnHL complex with a p*K*_a_ = 7.56, which corresponds to the deprotonation of a coordinated water molecule. The next form, ZnL, appears at pH around 8 and dominates in solution at pH range 8.4–9.9. A slight decrease of the p*K*_a_ value for Zn(ii) complex in comparison to the free ligand (8.45 and 8.80 respectively) suggests a deprotonation of the non-bonding N-terminal amine group. This mode of coordination also stays in the agreement with the NMR results, where signals for Phe1 remain unaltered, which proves that around pH 7.4, the N-terminal amine group does not participate in Zn(ii) binding. (Fig. S5†). The last deprotonation (pH around 9.5) results in the formation of ZnH_-2_L species and has no impact on the mode of coordination – most likely, the two lost protons come from the non-bound Lys and from the deprotonation of a water molecule, bound to the central Zn(ii) ion.

### Cu(ii) complexes

**Fig. 6:**
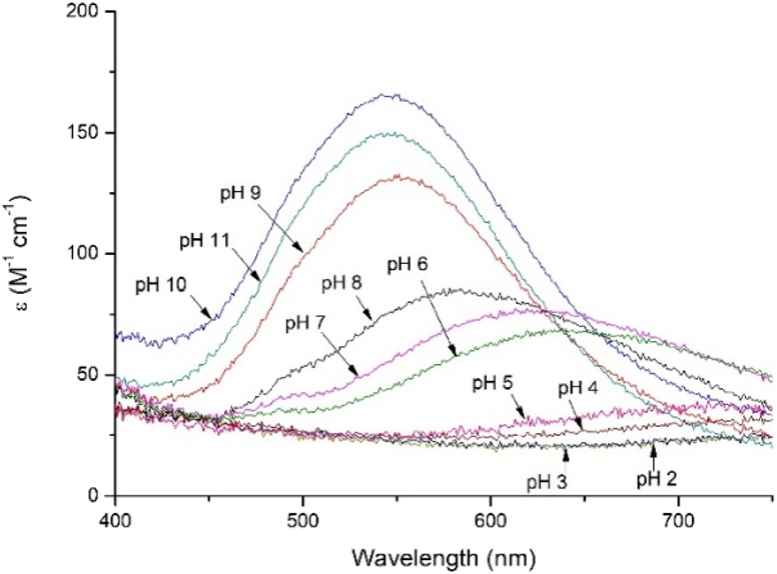
Electronic absorption spectroscopy in the UV-vis region of Cu(ii) complexes with PvHCt in pH range 2–11. Conditions: *T* = 298 K, *I* = 40 mM SDS, [Cu(ii)] = [PvHCt] = 0.001 M.

**Fig. 7:**
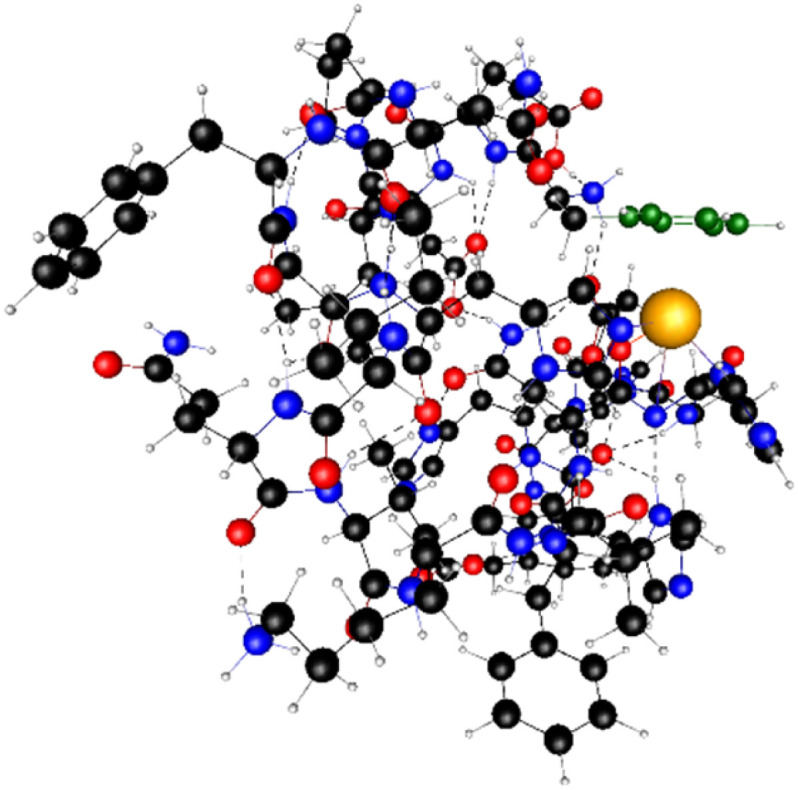
The binding pocket of the Cu(ii) cation with phenyl group of Phe7 (in green).

As in case of Zn(ii)-PvHCt, peptide forms only mononuclear complex with Cu(ii), with a metal:ligand ratio 1 : 1 (Fig. S1C and D†). The first complex observed at acidic pH, CuH_6_L (log β = 52.40), starts to form around pH 5 (Table 1 and Fig. S2B†). At this point, all acidic residues are deprotonated and Cu(ii) ion is bound by one imidazole nitrogen from the histidine residue. This coordination mode is also shown by the electronic absorption spectroscopy in the UV-vis region at pH 5 (Fig. 6), when a low, wide band with a maximum around 720 nm is observed.^32^ The next species, CuH_5_L, appear above pH 5.5. In the UV-vis region of the electronic absorption spectra (Fig. 6), the maximum of the band registered at pH 6 is significantly shifted towards shorter wavelengths (*λ*_max_ = 650 nm), which clearly indicates the coordination of the second nitrogen. The rapid appearance of the d–d band in the CD spectra at pH 6 (Fig. 7, S6B†), with a minimum at 605 nm points to the formation of the square planar complex,^33^ which in case of two coordinated nitrogens could be formed only in the {N_im_, N^−^} binding mode. Moreover, the p*K*_a_ value of CuH_5_L binding constant (6.02) also suggests that this species may correspond to coordination of amide nitrogen from the main chain, resulting in the {N_im_, N^−^} binding mode. Loss of the next proton leads to formation of the CuH_4_L complex, which occurs above pH 6 and is the most abundant one around 6.6.

The slight blue shift of the electronic absorption spectroscopy UV-vis band registered at pH 7 (Fig. 6, *λ*_max_ = 625 nm) suggests the involvement of the next imidazole nitrogen from a His residue, resulting in the {2N_im,_ N^−^} coordination mode.^32^

**Fig. 8:**
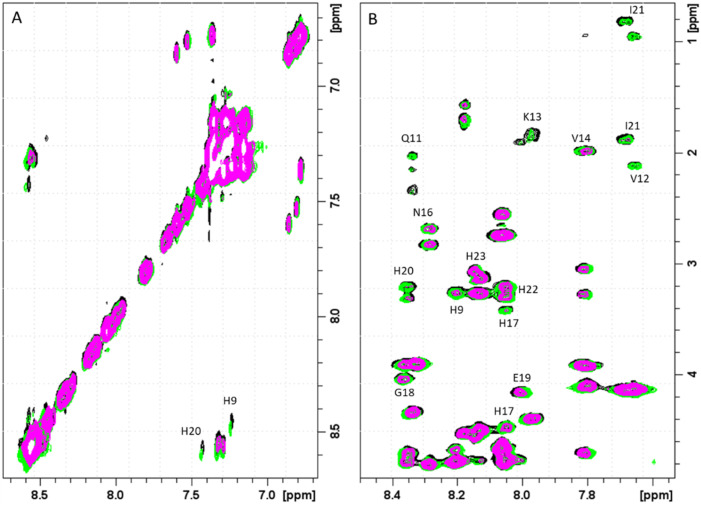
(A) Aromatic and (B) finger-print regions of 2D ^1^H-^1^H TOCSY spectra of PvHCt 0.5 mM, SDS-d_26_ 40 mM, pH 7.4, *T* 298 K in absence (black) and in presence of 0.1 Cu(ii) eqs (green) and 0.2 Cu(ii) eqs. (magenta). The most affected residues of the peptide sequence are shown.

Next four complex species (CuH_3_L, CuH_2_L, CuHL, CuL) correspond to deprotonation of three histidine residues (p*K*_a_ values of 6.57, 7.21 and 7.44 respectively) and N-terminal amine group (p*K*_a_ = 8.51), which does not take a part in the coordination. Its non-bonding character can be suggested not only by the small differences between p*K*_a_ values of ligand and corresponding complex form, but also by NMR results. As expected, the addition of the paramagnetic ions to PvHCt solutions caused selective line broadening on the NMR signals of protons close to the metal center.^34–37^ Correlations for Phe1 remain unaltered, which proves that around pH 7.4, the N-terminal amine group does not participate in Cu(ii) binding (Fig. S7†). Moreover, from Fig. 8, which reports the comparison of 2D ^1^H-^1^H TOCSY spectra of PvHCt in absence and in presence of two different Cu(ii) concentrations, it becomes evident that not all the His are equally affected by copper(ii) – two out of five H*δ*–H*ε* correlations are still visible in the NMR spectra (Fig. 8A). The ones disappearing belong to H9, H17 and H20 only, strongly indicating their participation in the metal coordination sphere. The involvement of H9, H17 and H20 in Cu(ii) binding is also supported by the line broadening detected in the ^1^H-^1^H TOCSY finger-print region (Fig. 8B). In fact, the largest variations induced by the paramagnetic ion are observed for the correlations mentioned above: H9, H17 and H20. Moreover, selective line broadening is observed on signals belonging to Q11, V12, K13, V14 and N16, that are residues present between H9 and H17. These variations, mostly located at the side-chain protons, might be explained by considering structural rearrangements on the α-helical region, encompassing the two copper binding residues H9 and H17. The Cu(ii)-induced changes on PvHCt structure are also in agreement with the CD data, which show significant differences before and after addition of Cu(ii) (Fig. 5) and with results of DFT calculations, which show precisely the same conclusions about the Cu(ii) binding mode – the metal forms 3N type connections with two imidazole rings of His9 (Cu–N 2.167 Å) and His17 (Cu–N 2.010 Å) supported by one amide N of His17 (Cu–N 2.511 Å) and one carbonyl – copper(ii) interaction (Cu–N 2.041 Å).

It is important to note that the copper complex is more compressed in comparison to the Zn(ii) ones – the distance of the most remote atoms in the Cu(ii) complex is close to 24 Å, while the average distance of the most remote atoms in the Zn(ii) complex equals about 30 Å. Another substantial difference in the metal complexes lies in the amount of their helical fragments. The ideal uncapped 23-residue ligand (without prolines) would be able to form 20 alpha-helical formamide-like hydrogen bond (HB) interactions that can support an alpha helix. In this case, the copper(ii) complex adopts a more helical structure of the ligand in comparison to the complexes with Zn(ii). We found 9 helical type HBs in the Cu(ii) complex, but only 6 in the A complex of the ZnL and 7 helical HBs both for B and C complexes of Zn(ii).

Also the comparison between the FTIR spectra of Zn(ii)-PvHCt and Cu(ii)-PvHCt complexes indicates a different structural rearrangement of the peptide backbone. As shown in Fig. S8,† the amide I region of the spectra exhibits different spectroscopic features depending on the metal ion coordination. In particular, the spectra of the two complexes differ in the absorptions around 1660–1650 and 1630 cm^−1^, typically associated with the presence of specific secondary structure elements. Specifically, the changes observed on the band at 1660–1650 cm^−1^ suggests an increase of α-helix conformation in the Cu(ii)-PvHCt system.^38–40^

Not only did 3 individual techniques confirm the same binding mode and the same type of structure; in addition, DFT revealed another very interesting issue here, which may contribute to the overall stability of the Cu(ii) complex by preventing the formation of polymorphic binding states – the phenyl group of Phe7 is located directly above the connected metal that “seals” copper in the binding pocket, as presented in Fig. 7.

The next two copper(ii) complexes that appear at pH above 8.5 and 9.5 (CuH_-1_L and CuH_-2_L, respectively), and correspond with the coordination of two amide nitrogens, resulting in the {2N_im_, 2N^−^} and finally, the {N_im_, 3N^−^} binding mode. This change of coordination mode is clearly visible in the UV-vis region of the electronic absorption spectra (Fig. 6), where bands for pH range 9–11 are significantly shifted towards shorter wavelengths. The maximum of the bands (548 nm) points to the coordination of the four nitrogens.^32^ The last form, CuH_-3_L corresponds to deprotonation of the Lys residue and has no impact on the coordination mode.

### Electrochemical properties

The redox behaviour of the PvHCt peptide by itself and in a complex with Cu(ii) was investigated by cyclic voltammetry in solution 4 mM HNO_3_/96 mM KNO_3_ at pH 7.4, 8.2 and 9, chosen on the basis of the species distribution diagram. Fig. S9† presents cyclic voltammograms recorded in the potential range of 0.2–0.95 V with *v* = 100 mV s^−1^. In the absence of copper ions, the PvHCt peptide exhibits an increasing current from 0.5 V. This wave shape curve can be ascribed to the oxidation of the His residues (H9, H17, H20, H22 and H23) based on the known electrochemical behaviour for this amino acid.^41^ For the Cu(ii)-PvHCt complex, at pH 7.4, a broad oxidation peak related to the Cu(ii)/(iii) process could be observed. No reduction signal corresponding to the Cu(iii)/Cu(ii) process was detected. The increase of the scan rate (*v* = 500 mV s^−1^, data not shown) did not result in the appearance of the reduction peak. The electrochemical response changed when experiments were performed at pH 8.2 where three histidines, H9, H17 and H20 participate in the metal coordination sphere. In the CV, both an anodic peak (*E*_a_ = 771 mV) and a cathodic peak (*E*_c_ = 692 mV) were present. The formal potential (*E*_f_ = (*E*_a_ + *E*_c_)/2) and peak separation (Δ*E*) were determined as 732 mV and 79 mV, respectively. Since the oxidation current is higher than that of the reduction current, this process should be labelled as quasi-reversible. The lower current for the cathodic process Cu(iii)/Cu(ii) process can be explained by rapid oxygen-induced decarboxylation followed by hydroxylation at the carboxyl end of the peptide, occurring *via* a Cu(iii) intermediate.^42^ The electrochemical response for the tested complex at pH 9 was almost identical to that obtained at pH 8.2 (see Fig. S10†).

CVs recorded at lower potentials, from 0.4 V up to −0.4 V (Fig. S11†) at pH 7.4 revealed an irreversible Cu(ii)/(i) redox process with a slow electron transfer (separation between cathodic (*E*_c_ = – 70 mV) and anodic (*E*_a_ = 90 mV) peaks was *ca.* 160 mV). This low-potential reaction could be connected with the formation of harmful ROS under biologically relevant conditions because of the formal potential (*E*_f_) 10 mV *vs.* Ag/AgCl.^43,44^ Additionally, the electrochemical behaviour of Cu(ii) bound to the PvHCt peptide is strongly influenced by the geometrical structure of the formed complexes because both signals started to disappear at higher pH (Fig. S11† red curve).

### Antimicrobial activity

The antimicrobial properties of PvHCt peptide and its Zn(ii) and Cu(ii) complexes were tested against several bacterial strains (*E. faecalis*, MRSA, *E. coli*, *K. pneumoniae*, *A. baumannii and P. aeruginosa*) and fungi (*C. albicans*). Obtained results are presented in Table 3; detailed dose dependence of the antimicrobial tests for PvHCt and its metal-complexes is shown in Table S1.†

**Table 3:** *In vitro* antibacterial activity of PvHCt peptide and its Zn(ii) and Cu(ii) complexes, determined as a minimal inhibitory concentration required to inhibit the growth of 50% microorganisms (MIC50) (μg mL^−1^); n/d, not determined. Experiments were performed according to the ISO 20776–1:2019 (ref. 45) and ISO 16256:2012.^46^ No MIC50 values were determined for *P. aeruginosa*, *K. pneumoniae*, *A. baumannii* and *C. albicans* and are therefore not included. No MBC/MFC activity was observed after performing modified Richard's method

	*Escherichia coli*	MRSA	*Enterococcus faecalis*
	MIC50 [μg mL^−1^]	MIC50 [μg mL^−1^]	MIC50 [μg mL^−1^]
PvHCt	n/d	n/d	n/d
Zn(ii)-PvHCt	n/d	32	512
Cu(ii)-PvHCt	16	16	16

Antibacterial activity (MIC50) of the PvHCt peptide is only induced by the presence of metal ions in the solution – quite surprisingly, PvHCt alone has no antimicrobial activity on the pathogen strains tested in this work and only the formation of the Zn(ii) or Cu(ii) complex enabled the inhibition of the growth of microorganisms.

Moreover, the Cu(ii)-PvHCt complex has significantly higher antimicrobial activity than the complex with Zn(ii).

Potential cytotoxicity of antimicrobial active peptide-metal systems were also checked. Results included in Table S2† prove that the analysed compounds exhibit cytotoxic activity at a concentration of 75 μM, limiting their application potential to the above mentioned concentration. Also control experiments with metal salts only were carried out in order to confirm that the observed effect was due to the activity of the complex, and not to the antimicrobial activity of the metal ions alone (Table S3†).

It is absolutely intriguing to find out which feature of the complexes is triggering their antimicrobial activity – it is certainly not an effect of Cu(ii) withdrawal from the pathogen, as the Cu(ii)-PvHCt complexes are not of exceptional stability (Fig. S12†).

Is it the structural change that occurs after metal binding? As mentioned before, at pH 7.4 both the peptide and the complexes present a partially α-helical structure (Fig. 5). In case of the Zn(ii) complex, a high similarity with the spectrum of the free peptide is observed, which suggests that the metal binding did not significantly affect the secondary structure of the system. At the same time, the complex with copper(ii) differs noticeably from the rest. The analysis of the secondary structure (Table S4†) carried out by various computational programs^47,48^ suggests that the observed differences probably correspond to the increased percentage of the α-helical structure, which is consistent with the trend observed in NMR and FTIR spectroscopies and further confirmed by DFT calculations. A moderate increase in the proportion of helical structure, together with a conformational change of the complex caused by the coordination of a His9 residue (distant from His17, the other binding site) most likely contribute to the significantly increased antimicrobial activity.

## Conclusions

The coordination of copper(ii) to PvHCt, a novel antimicrobial shrimp peptide, strongly impacts its structure and antimicrobial properties, showing a clear and intriguing link between metal coordination, structure and function. It is indeed fascinating to see that PvHCt inhibits the growth of microorganisms only in the presence of metal ions. Around pH 7.4, PvHCt binds Zn(ii) to its C-terminal part *via* 2 of the available histidines: (i) His20 and His23, (ii) His22 and His23, or (iii) His17 and His22. This type of binding does not significantly affect the secondary structure of the peptide (Fig. 5), but it does, to some extent, influence its antimicrobial properties – the Zn(ii)-PvHCt complex becomes active against MRSA, while the PvHCt peptide itself shows no activity against any of the tested human pathogens.

The most interesting phenomenon is observed in the case of Cu(ii)-PvHCt complexes – Cu(ii) binds to the central part of the peptide, to His9 and His17, inducing a significant structural change, enhancing the percentage of α-helix and triggering ROS formation. This has a very pronounced effect on the antimicrobial activity of the complex, making it active against *E. coli*, MRSA and *E. faecalis*, with quite a promising MIC of 16 μg mL^−1^. We expect that the structural difference observed for Cu(ii)-PvHCt (due to the coordination of His9 and His17, located quite far from each other in the sequence) is most likely the trigger of its antimicrobial activity – it induces a pronounced structural change and also increases the content of α-helical structure, which can often be attributed to the improvement of membranolytic properties. In addition, an irreversible, slow and low potential Cu(ii)/(i) process takes place, which could be connected with the formation of harmful ROS under biologically relevant conditions and could additionally contribute to the increase of antimicrobial potency. This Cu(ii) reducing behaviour is strongly influenced by the geometrical structure of the complex, because this phenomenon is no longer observed at higher pH, where more amides participate in binding.

It again becomes clear that there is a significant and underestimated effect of metal coordination on the activity of antimicrobial peptides. The structural change of the discussed copper(ii) complex triggers a different mode of action, initiating the antimicrobial properties of AMPs.

We have recently described a similar effect of metal-triggered structural change of the antidiabetic (and also antifungal) pramlintide, where Zn(ii) binding to the N-terminal amine group and to the imidazole of His18 resulted in a kink of the peptide, which triggered the formation of fibrils;^49^ changes in the structure also enhanced an antimicrobial activity in case of the Zn(ii) complex with shepherin, an AMP derived from the plant *Capsella bursa-pastoris*.^50^

To the best of our knowledge, this is the first work which describes a Cu(ii)-induced structural rearrangement of an antimicrobial peptide, which leads to increased ROS production and triggers its antimicrobial potency. The Cu(ii)-PvHCt complex may become a promising antimicrobial agent; that is why further studies aiming to elucidate the precise underlying mechanisms of its mode of action are strongly encouraged.

## Experimental

### Materials

The FEDLPNFGHIQVKVFNHGEHIHH peptide was purchased from KareBay Biochem (USA) (certified purity: 98%) and was used as received. The carbonate-free stock solutions of 0.1 M NaOH were purchased from Sigma-Aldrich and then potentiometrically standardized with the primary standard potassium hydrogen phthalate (99.9% purity).

### Mass spectrometry

High-resolution mass spectra were obtained on a Bruker Apex Ultra FT-ICR (Bruker Daltonik, Bremen, Germany), equipped with an Apollo II electrospray ionization source with an ion funnel. Mass spectrometer was operated in the positive io mode. The instrumental parameters were as follows: scan range *m*/*z* 100–2000, dry gas–nitrogen, temperature 473 K, and ion energy 5 eV. The capillary voltage was optimized to the highest S/N ratio and it was 4200 V. The samples were prepared in a 1 : 1 methanol–water mixture with a M(II): L molar ratio 1 : 1, [ligand] = 3 × 10^−4^ M, pH 6. The samples were infused at a flow rate of 3 μL min^−1^. The instrument was calibrated externally with a Tunemix™ mixture (Bruker Daltonik, Germany) in quadratic regression mode. Data was processed using the Bruker Compass DataAnalysis 4.0 program. The mass accuracy for the calibration was better than 5 ppm, enabling together with the true isotopic pattern (using SigmaFit) an unambiguous confirmation of the elemental composition of the obtained complex.

### Potentiometric titration

Stability constants for proton, Zn(ii) and Cu(ii) complexes of the peptides were calculated from pH-metric titration curves obtained over the pH range 2–11 at *T* = 298 K in water solution of 4 mM HClO_4_ and ionic strength 40 mM (SDS), using a total volume of 3 mL. The potentiometric titrations were performed using a Metrohm Titrando 905 titrator and a Mettler Toledo InLab Micro combined pH electrode. The thermostabilized glass-cell was equipped with a magnetic stirring system, a microburette delivery tube and an inlet–outlet tube for argon. Solutions were titrated with 0.1 M carbonate-free NaOH. The electrodes were calibrated daily for hydrogen ion concentration by titrating HClO_4_ with NaOH under the same experimental conditions as above. The purities and exact concentrations of the peptide solutions were determined by the Gran method.^51^ The peptide concentration was 0.5 mM and the Zn(ii)- and Cu(ii)-to-peptide ratio was 1 : 1. The standard potential and the slope of the electrode couple were computed by means of the GLEE program.^52^ The HYPERQUAD 2006 (ref. 30) program was used for the stability constant calculations. The standard deviations were computed by HYPERQUAD 2006 and refer to random errors only. The constants for the hydrolytic Zn(ii) species were used in these calculations. The distribution and competition diagrams were computed with the HYSS program.^53^

### Spectroscopic methods

The absorption spectra were recorded on Varian Cary 300 Bio spectrophotometer, in the range 200–800 nm, using a quartz cuvette with an optical path of 1 cm. Circular dichroism (CD) spectra were registered on a Jasco J-1500 CD spectrometer in the 200–800 nm range, using a quartz cuvette with an optical path of 1 cm or with a cuvette with an optical path of 0.01 cm in the wavelength range 180–300 nm. The solutions were prepared in a water solution of 4 mM HClO_4_ at ionic strength *I* = 40 mM (SDS). The peptide concentration was 1 mM (for electronic absorption spectroscopy and circular dichroism in 200–800 nm range) or 0.1 mM (for CD in 180–300 nm range) and the Cu(ii)-to-peptide ratio was 1 : 1.

FTIR spectra were recorded using an Agilent Cary 630 spectrometer equipped with an attenuated total reflectance (ATR) FTIR technique. Peptide solutions were applied as two microliter droplets onto the ATR crystal and allowed to dry slowly at room temperature, forming thin hydrated films. The spectra were collected at a resolution of 4 cm^−1^, employing a diamond ATR (5 Bounce ZnSe ATR and DialPath/TumblIR). The measurements were taken in the mid-IR region (approximately 4000–800 cm^−1^) with 128 co-added scans at a spectral resolution of 4 cm^−1^. All spectra were presented in absorption mode and smoothed using the Savitzky–Golay algorithm.

### NMR experiments

NMR experiments were conducted at a magnetic field strength of 14.1 T on a 600 MHz Bruker Avance III Spectrometer. All the experiments were collected at 298 K, employing a 5 mm SEI (sensitive enhancement) probe. Chemical shifts were calibrated using the external reference 3-(Trimethylsilyl)-propionic-2,2,3,3-*d*_4_ acid sodium salt (TMSP-*d*_4_). Standard pulse sequences were applied to record TOCSY and NOESY spectra. In order to optimize the NOE transfer, NOESY spectra were acquired with a mixing time varying between 100 and 300 ms. NMR spectra were processed with TopSpin 3.6 software and analysed with the program Sparky.^54^ Suppression of residual water signal was achieved by excitation sculpting,^55^ using a selective 2 ms long square pulse on water. The peptide was dissolved in SDS-*d*_25_ 40 mM aqueous solutions (90 : 10H_2_O : D_2_O) to obtain a final concentration 0.5 mM. The pH was adjusted by adding small aliquots of NaOH and HCl to reach a final value around 7.4. TMSP-*d*_4_ was used as internal reference for chemical shift of ^1^H NMR resonances. The NMR assignment of the peptide was obtained by the analysis of ^1^H-^1^H 2D TOCSY and NOESY spectra and it is reported in Table S5.†

### Electrochemical methods

Electrochemical experiments were performed on glassy carbon disk (Ø 3 mm, Mineral) electrodes (GCE) using an Autolab PGSTAT204 potentiostat (Metrohm AG) controlled by the NOVA software (version 2.1.6). Cyclic voltammetry (CV) measurements were performed at different scan rates, and at least 3 scans were recorded for each series. The acquisition of voltammetric curves was repeated at least 3 times for each solution of PvHCt-Cu(ii) complex and PvHCt peptide. All experiments were performed in a three-electrode arrangement, with the GCE working electrode, a Ag|AgCl|3 M NaCl reference electrode, and a platinum rod as the counter electrode. The reference electrode was separated from the working solution by a salt bridge filled with a 0.1 M KNO_3_ solution, at the same pH as in the cell. The potential of the reference electrode was calibrated based on the ruthenium electrode process. The formal potential of hexaammineruthenium(iii/ii) chloride in 0.5 M KNO_3_ is 0.172 ± 0.002 V *vs.* Ag/AgCl. Prior to each voltammetric measurement, the GCE was polished on a Buehler polishing cloth to a mirror-like surface, using aqueous slurries of 1 and 0.3 μm alumina powder followed by 1 min water ultrasonication to remove the remaining powder. All electrochemical measurements were carried out in a 96 mM KNO_3_ solution containing 4 mM HNO_3_. The pH was adjusted by adding a small volume of concentrated KOH and HNO_3_ solutions. Based on the distribution diagram, we selected three pH values at which the experiments were carried out: pH 7.4 (corresponding to physiological pH), pH 8.2, where the CuHL form predominates, and 9.0, where the CuL form predominates. The pH was closely controlled before, during and at the end of each voltammetric measurement. The concentrations of the metal-free PvHCt peptide and in complexes were 0.5 mM. The ligand-to-copper(ii) ratio was 1 : 0.9 in all cases (a small deficiency of Cu(ii) helps to avoid interference from uncomplexed Cu(ii) cations).

### DFT calculations

Molecular orbital studies on Cu(ii) and Zn(ii) 1 : 1 complexes with FEDLPNFGHIQVKVFNHGEHIHH have been done on the DFT level of theory. The starting structure of the peptide for DFT calculations was generated on the basis of the amino acid sequence after 75 ps simulation at 300 K, without cutoffs using BIO+ implementation of CHARMM force field. DFT calculations were performed with Gaussian 16C.01 (ref. 56) suite of programs using the ωB97X-D^57^ long-range corrected hybrid density functional with damped atom–atom dispersion corrections was used with a double-*ζ* 6-31G(*d*,*p*) basis set containing polarization functions. All presented structures were fully optimized. All presented complexes are thermodynamically stable.

### Antimicrobial activity assay of peptide and peptide-metal ion complex system

Seven reference strains from ATCC collection (*Acinetobacter baumannii* (ATCC 19606), *Klebsiella pneumoniae* (ATCC 700603), *Pseudomonas aeruginosa* (ATCC 27853), *Escherichia coli* (ATCC 25922), *Staphylococcus aureus* MRSA (ATTC 43300), *Enterococcus faecalis* (ATCC 29212) and *Candida albicans* (ATCC 10231)) were used for antimicrobial activity assay. The antimicrobial effect of analysed peptides/complexes and control experiments with metal ions only were performed according to the standard protocol using microdilution method with spectrophotometric measurement (*λ* = 580 nm at starting point and after 24 h) according to the ISO standard 20776-1:2019, ISO standard 16256:2012 and modified Richard's method.^45,46,58,59^ Stock peptide solutions were prepared in deionized sterile water four times concentrated. Serial dilutions of ligand/complex solution were made on 96-well microplates in the range between 0.5 μg mL^−1^ and 256 μg mL^−1^. Only for *Enterococcus faecalis* (ATCC 29212), the concentration range has been expanded until 1024 μg mL^−1^, due to the probability of MIC50 at the end of the first analysed range. Tryptone Soya Agar (TSA) plates were inoculated with microbial strains from glycerol stocks. After 24 h/37 °C incubation (for bacteria) or 24 h/25 °C (for fungus), a proper density of bacterial and fungal suspension was prepared using a densitometer (final inoculum (5 × 10^5^ CFU mL^−1^ for bacteria and 0,5–2,5 × 10^5^ CFU mL^−1^ for fungus) in Tryptic Soy Broth (TSB)). A positive (TSB + strain) and negative control (TSB) were also included in the test. Spectrophotometric solubility control of each peptide and peptide-metal ion system was also performed. For each strain, the validation process was performed using following antibacterial/antifungal agents: levofloxacin, gentamicin, amphotericin B, according to the EUCAST examination. Obtained bactericidal/fungicidal concentrations were for *A. baumannii* (ATCC 19606): levofloxacin 0.5 μg mL^−1^, *E. coli* (ATCC 25922): gentamicin 4 μg mL^−1^, *E. faecalis* (ATCC 29212): levofloxacin 4 μg mL^−1^, *P. aeruginosa* (ATCC 27853): levofloxacin 1 μg mL^−1^, *S. aureus* MRSA (ATCC 43300): levofloxacin 1 μg mL^−1^, *K. pneumoniae* (ATCC 700603): gentamicin 4 μg mL^−1^, *C. albicans* (ATCC 10231): amphotericin B 1 μg mL^−1^. Microplates were incubated at 37 ± 1 °C or 25 ± 1 °C for 24 hours on the shaker (500 rpm). After this, the spectrophotometric measurement was performed at 580 nm. The minimal inhibitory concentration required to inhibit the growth of 50% microorganisms (MIC50) was determined as the lowest concentration of an antimicrobial agent that decreased the measured microbial growth to 50% as referred to positive control. Then 50 μL aliquots of 1% (m/v) 2,3,5- triphenyltetrazolium chloride (TTC) solution were added into each well. TTC is a chemical indicator which is converted into red formazan crystals in living microbial cells. MBC/MFC can be observed as the lowest concentration required to kill a particular microbial strain, determined by visual analysis after 24 h incubation with TTC (did not change the colour to pink). Thanks to both methods, MIC50 and MBC or MFC can be determined.

### Neutral red cytotoxicity assay

For each peptide and peptide-metal ion system, where the antimicrobial activity was determined, a Neutral Red (NR) cytotoxicity assay was performed using human primary renal proximal tubule epithelial cells (RPTEC) from ECACC collection. The experiment was performed according to ISO 10993-5:2009 (ref. 60) and ISO/IEC 17025:2005.^61^ A standard protocol for the NR assay was used from Nature Protocol.^62^ MEMα supplemented with 10% FBS, 2 mM l-glutamine and suitable amount of antibiotics (amphotericin B, gentamycin) was used for the experiment. Also Cu(ii) and Zn(ii) salt solutions were checked to eliminate potential cytotoxic effect of metal ions. Stock peptide solutions were prepared in deionized sterile water and then 100 times diluted in the medium. After adding proper mixtures of testing compounds and cells (1 × 10^4^) into each well, plates were incubated for 48 and 72 h in 5% CO_2_ at 37 °C. Next, medium was removed and 100 μL of NR solution (40 μg mL^−1^) was added to each well followed by incubation for 2 h at 37 °C. After removing the dye, wells were rinsed with PBS and left to dry. Then, NR destain solution (1% glacial acetic acid, 50% of 96% ethanol and 49% of deionized water; v/v) was added to each well. The plates were shaken (30 min, 500 rpm) until NR was extracted from the cells and formed a homogenous solution. The absorbance was measured using microplate reader at 540 nm. As a negative control untreated cells were considered as 100% of potential cellular growth. Furthermore, cells incubated with 1 μM staurosporine were used as a positive control.

## Author contributions

The manuscript was written through the contributions of all authors. All authors have given approval to the final version of the manuscript.

## Conflicts of interest

There are no conflicts to declare.

## Supplementary Material

SC-016-D4SC05222F-s001

## Data Availability

The data supporting the article “Cu(ii) binding to an antimicrobial shrimp peptide – a small step for structural chemistry, a big leap for medicinal applications” have been included as part of the ESI.† If any additional questions regarding experimental details may arise, the corresponding authors remain at the Readers' disposal.
