# Supplementary material for: Cu(ii) binding to an antimicrobial shrimp peptide – a small step for structural chemistry, a big leap for medicinal applications
Source: Chem Sci. 2025 Jan 23;16(8):3447–58. doi: 10.1039/d4sc05222f (PMC11775653; doi:10.1039/d4sc05222f)
Supplement: SC-016-D4SC05222F-s001 [file SC-016-D4SC05222F-s001.pdf]

Supplementary Information (SI) for Chemical Science.
This journal is © The Royal Society of Chemistry 2025

Cu(II) binding to an antimicrobial shrimp peptide - a small step for structural chemistry, a big leap for medicinal applications

Adriana Miller,[a] Agnieszka Matera-Witkiewicz,[b] Aleksandra Mikołajczyk,[b] Arian Kola,[c] Magdalena Wiloch,[d] Martin Jonsson-Niedziolka,[d] Robert Wieczorek,[a] Joanna Wątły,[a] Daniela Valensin*[c] and Magdalena Rowińska-Żyrek*[a]

[a] *Faculty of Chemistry, University of Wrocław, ul. F. Joliot-Curie 14, 50-383 Wrocław, Poland*
[b] *Screening of Biological Activity Assays and Collection of Biological Material Laboratory, Wrocław Medical University Biobank, Faculty of Pharmacy, Wrocław Medical University, ul. Borowska 211A, 50-556 Wrocław, Poland*
[c] *Department of Biotechnology, Chemistry and Pharmacy, University of Siena, via Aldo Moro 2, 53100 Siena, Italy*
[d] *Institute of Physical Chemistry, Polish Academy of Sciences, ul. Marcina Kasprzaka 44/52, 01-224 Warszawa*
Corresponding Author
* Daniela Valensin; e-mail: valensindan@unisi.it
*Magdalena Rowińska-Żyrek; e-mail: magdalena.rowinska-zyrek@uwr.edu.pl

Electronic Supplementary Information (ESI)

Table S1: Dose dependence of the antimicrobial tests for PvHCt and its Cu(II) and Zn(II) complexes.

Concentration	256 µg/ml	128 µg/ml	64 µg/ml	32 µg/ml	16 µg/ml	8 µg/ml	4 µg/ml	2 µg/ml	1 µg/ml	0,5 µg/ml
**Viability %**										
***S. aureus* MRSA**										
**PvHCt**	93	93	92	99	100	98	98	102	102	104
**Zn(II)-PvHCt**	38	39	41	49	99	99	100	100	101	102
**Cu(II)-PvHCt**	4	5	6	5	50	61	88	91	93	94
P. aeruginosa										
**PvHCt**	80	80	80	80	80	80	84	90	96	96
**Zn(II)-PvHCt**	81	80	81	84	84	86	88	93	93	94
**Cu(II)-PvHCt**	72	73	79	78	79	79	87	88	99	99
K. pneumoniae										
**PvHCt**	84	92	92	92	92	93	93	93	93	93
**Zn(II)-PvHCt**	94	94	94	94	95	95	95	95	95	96
**Cu(II)-PvHCt**	81	87	93	93	91	91	89	90	94	96
A. baumannii										
**PvHCt**	84	85	85	85	85	85	85	89	93	100
**Zn(II)-PvHCt**	92	95	95	95	90	92	94	95	95	95
**Cu(II)-PvHCt**	84	85	85	85	85	85	85	87	98	98
C. albicans										
**PvHCt**	93	86	84	87	89	90	89	93	100	98
**Zn(II)-PvHCt**	90	93	94	95	95	95	92	98	100	100
**Cu(II)-PvHCt**	90	90	89	92	91	94	92	89	97	97
E. coli										
**PvHCt**	95	100	90	90	100	100	100	96	95	100
**Zn(II)-PvHCt**	70	93	94	95	95	95	100	100	102	105
**Cu(II)-PvHCt**	13	13	13	13	53	73	78	79	79	100

Concentration	1024 µg/ml	512 µg/ml	256 µg/ml	128 µg/ml	64 µg/ml	32 µg/ml	16 µg/ml	8 µg/ml	4 µg/ml	2 µg/ml	1 µg/ml	0,5 µg/ml
Enterococcus faecalis												
PvHCt	60	60	60	60	58	59	65	65	72	73	89	99
Zn(II)-PvHCt	40	50	54	64	65	65	86	87	94	98	100	100
Cu(II)-PvHCt	5	6	7	7	7	8	51	65	69	73	81	89

Table S2: Cell viability using Neutral red uptake assay (NR) performed on human primary renal proximal tubule epithelial cells (RPTEC) from ECACC collection, after 48 and 72 h incubation with PvHCt and PvHCt-metal ion systems.

	**Concentration [μg/mL]**	**Concentration [μM]**	**Incubation time**	
			48h	72h
			viability [%]	
**PvHCt**	344	125	53	0
	206.40	75	57	33
	27.52	10	94	86
	2.75	1	94	86
**Cu(II)-PvHCt**	351.94	125	62	22
	211.16	75	67	27
	28.16	10	101	86
	2.82	1	105	92
**Zn(II)-PvHCt**	352.18	125	47	2
	211.31	75	48	33
	28.17	10	87	85
	2.82	1	87	86

Table S3: *In vitro* antibacterial activity of Zn(II) and Cu(II) chlorides, determined as a minimal inhibitory concentration required to inhibit the growth of 50% microorganisms (MIC50) (μg/mL); n/d, not determined. % of viability were obtained after 24h of experiment.

Strain (ATCC)		% of viability in max. concentration (ug/mL)	% of viability in min concentration (ug/mL)	MIC (ug/mL)	MBC/MFC (ug/mL)
A. baumannii	Cu(II)	97%	95%	512	1024
	Zn(II)	96%	100%	n/d	n/d
E. coli	Cu(II)	94%	95%	1024	n/d
	Zn(II)	92%	96%	1024	n/d
E. faecalis	Cu(II)	82%	92%	1024	n/d
	Zn(II)	76%	90%	n/d	n/d
P. aeruginosa	Cu(II)	77%	95%	512	1024
	Zn(II)	80%	92%	1024	n/d
***S. aureus* MRSA**	Cu(II)	87%	92%	1024	n/d
	Zn(II)	86%	97%	1024	n/d
K. pneumoniae	Cu(II)	77%	95%	512	n/d
	Zn(II)	80%	100%	1024	n/d
C. albicans	Cu(II)	90%	86%	n/d	n/d
	Zn(II)	80%	92%	n/d	n/d

Table S4: Results of the analysis of secondary structure, obtained from BestSel (*1*) and DichroWeb (*2*) programs. Addition of Cu(II) ions causes a slight increase in percentage of helical structure.

PvHCt (peptide)		Cu(II)-PvHCt			
BestSel					
helix1+helix2	22,9 %	helix1+helix2	24,5 %	difference:	1,6 %
strand1+strand2	11 %	strand1+strand2	12,2 %		1,2 %
turns	3,7 %	turns	3,1 %		-0,6 %
unordered	44,8 %	unordered	38,6 %		-6,2 %
DichroWeb (Selcon3)					
helix1+helix2	26,2 %	helix1+helix2	30,2 %	difference:	4 %
strand1+strand2	23,4 %	strand1+strand2	21,3 %		-2,1 %
turns	23,1 %	turns	22,0 %		-1,1 %
unordered	28,9 %	unordered	28,4 %		-0,5 %

Table S5: Proton assignment of PvHCt 0.5 mM in SDS 40 mM, T=298 K, pH=7.4

	H_N	H_α	H_β	H_γ	Others
Phe-1		4.32	3.34-3.10		H_δ 7.37
Glu-2	8.68	4.36	2.09-1.90	2.28	
Asp-3	8.07	4.66	2.74		
Leu-4	8.18	4.52	1.79-1.70	1.58	H_δ 0.96-0.92
Pro-5		4.31	2.19-1.94	1.82-1.50	H_δ 3.78-3.49
Asn-6	8.07	4.63	2.73-2.55		H_δ 7.59-6.86
Phe-7	8.15	4.56	3.25- 3.06		H_δ 7.27
Gly-8	8.33	3.90			
His-9	8.21	4.66	3.25		H_δ 7.22
Ile-10	7.80	4.08	1.98	1.54-1.19	H_δ 0.93
Gln-11	8.34	4.33	2.14-2.02	2.31	H_δ 7.36-6.78
Val-12	7.66	4.13	2.14	0.95	
Lys-13	7.97	4.38	1.87-180	1.47	H_δ 1.73; H_ε 3.02
Val-14	7.68	3.90	1.99	0.81-0.67	
Phe-15	7.81	4.70	3.38-3.04		H_δ 7.36
Asn-16	8.27	4.81	2.82-2.68		H_δ 7.52-6.81
His-17	8.05	4.48	3.40-3.26		H_δ 7.33
Gly-18	8.36	4.04-3.91			
Glu-19	8.01	4.13	1.92	2.19	
His-20	8.35	4.71	3.30-3.22		H_δ 7.41
Ile-21	7.68	4.14	1.87	1.42-1.16	H_δ 0.81
His-22	8.06	4.73	3.28-3.20		H_δ 7.31
His-23	8.11	4.49	3.26-3.13		H_δ 7.29

Stoichiometry

Mass spectrometry indicated the stoichiometry of the formed PvHCt Zn(II) and Cu(II) complexes.

Fig. S1A and S1B present the MS spectra for Zn(II)-PvHCt complex. In the Fig. S1A, three main signals can be observed. The most intensive one (*m/z* = 688.8, *z* = 4+) comes from the single peptide and the other two were identified as a sodium adduct (*m/z* = 694.3, *z* = 4+) and Zn(II)-PvHCt complex (*m/z* = 704.3, *z* = 4+).

Fig. S1C and S1D show spectra for Cu(II)-PvHCt complex. In the Fig. S1C, eight signals can be observed, and the most intensive were assigned to a single peptide (*m/z* = 688.8, *z* = 4+), its sodium adduct (*m/z* = 694.3, *z* = 4+) and Cu(II)-PvHCt complex (*m/z* = 704.3, *z* = 4+).

In the Fig. S1B and S1D the experimental and simulated isotopic patterns were compared. The results confirm that PvHCt forms Cu(II) and Zn(II) complexes only in a mononuclear form, with a metal:ligand ratio 1:1.

A)

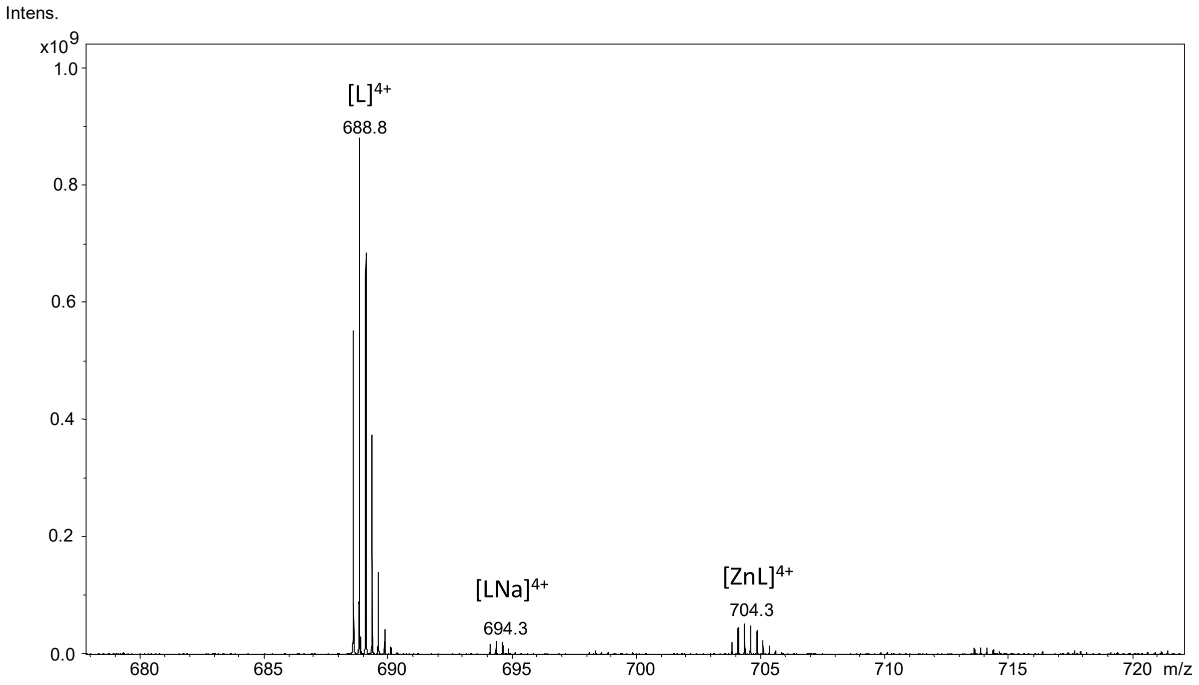

B)

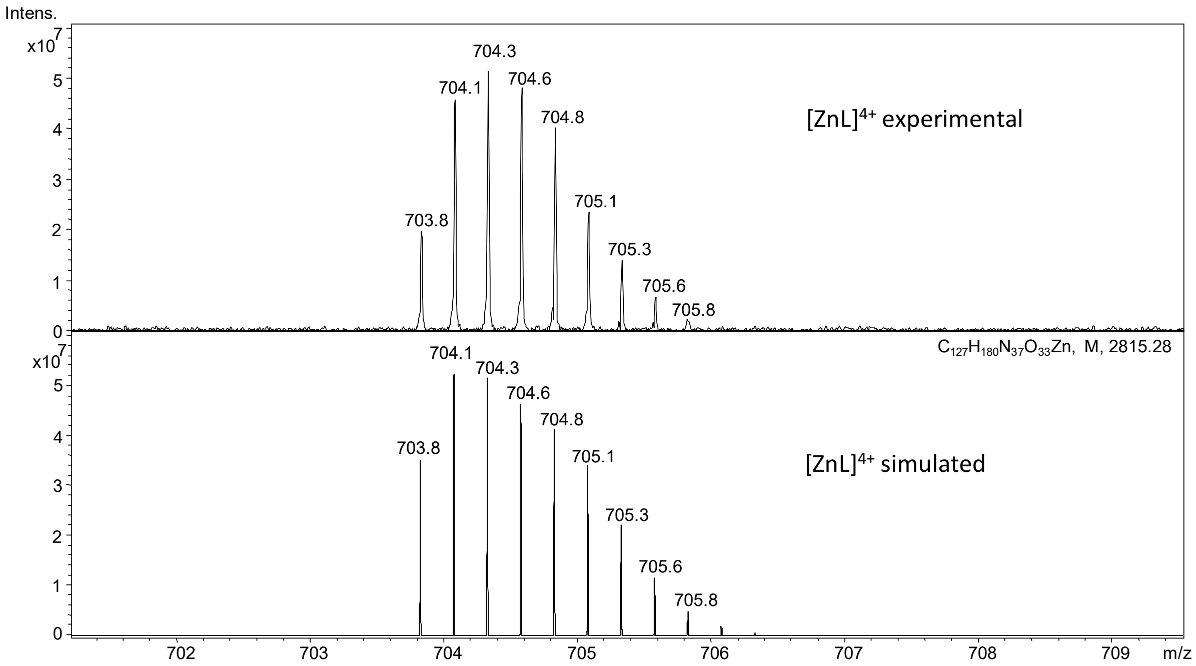

Intens.
x10^7
703.8
704.1
704.3
704.6
704.8
705.1
705.3
705.6
705.8
[ZnL]4+ experimental
C127H180N37O33Zn, M, 2815.28
[ZnL]4+ simulated
m/z

C)

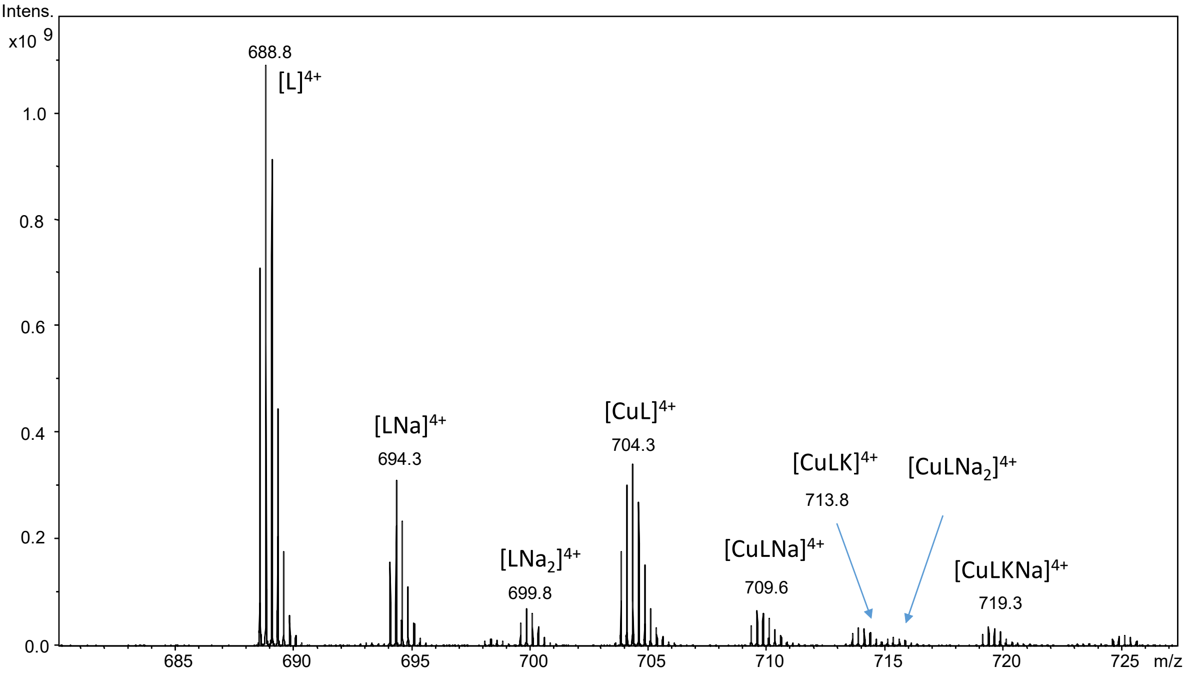

Intens.
x10 9
688.8
[L]4+
[LNa]4+
694.3
[LNa2]4+
699.8
[CuL]4+
704.3
[CuLNa]4+
709.6
[CuLK]4+
713.8
[CuLNa2]4+
[CuLKNa]4+
719.3
m/z

D)

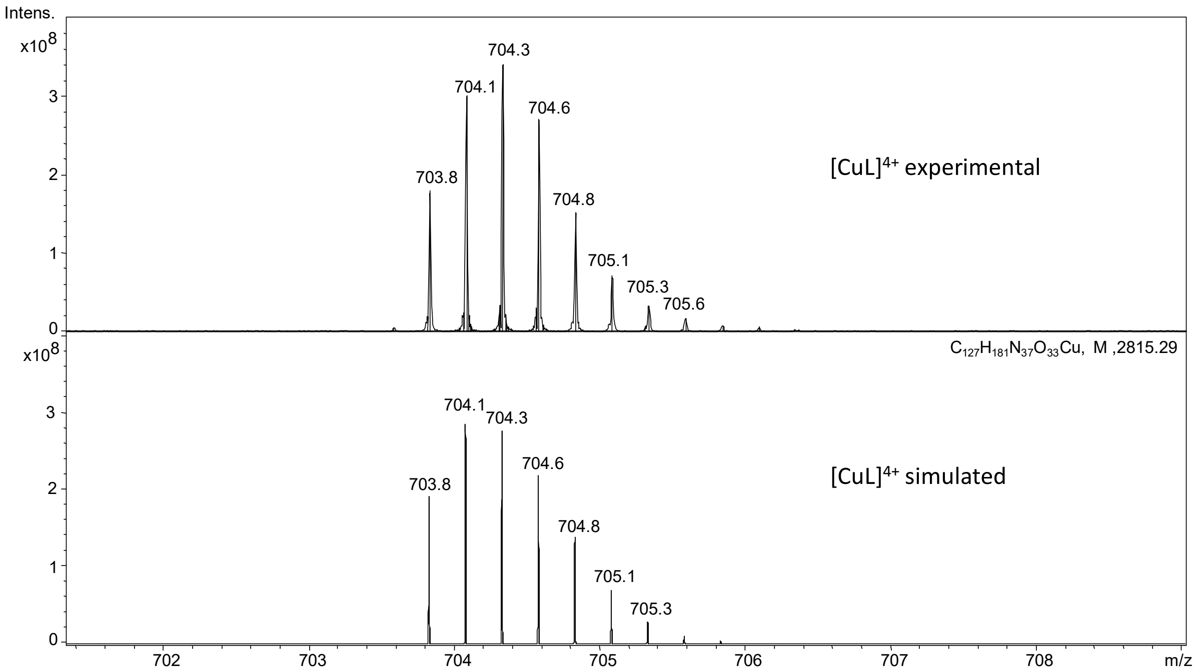

Fig. S1: ESI-MS spectra of: MS spectra of Zn(II)-PvHCt (A, B) and Cu(II)-PvHCt (C, D) samples. In the B and D, the experimental (top) and simulated (bottom) results are compared and clearly show the presence of the complex. Conditions: [Zn(II)] = [Cu(II)] = [PvHCt] = 3 x 10^{-4} M in a 1:1 methanol-water mixture; M^{2+}:peptide ratio was 1:1, pH = 6

A)

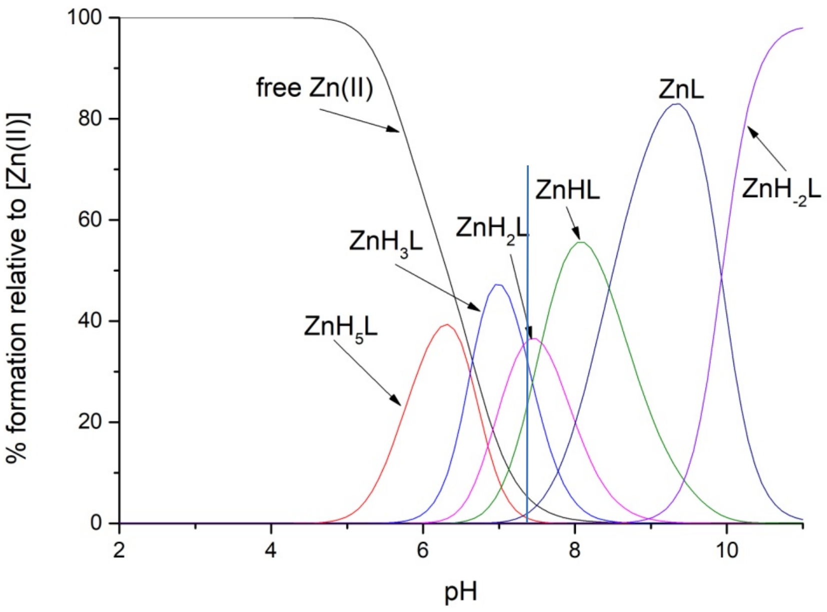

B)

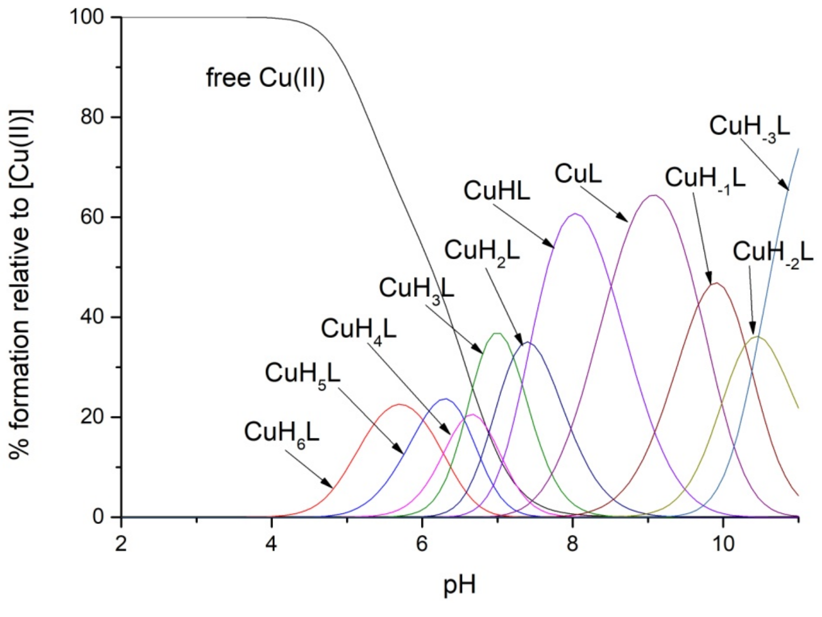

Fig. S2: Distribution diagrams for the formation of: A) Zn(II) complex with PvHCt; B) Cu(II) complex with PvHCt; T=298 K, *I*= 40 mM SDS, $[M^{2+}]=0.5\cdot10^{-3}$ M; M^{2+}:L molar ratio = 1:1

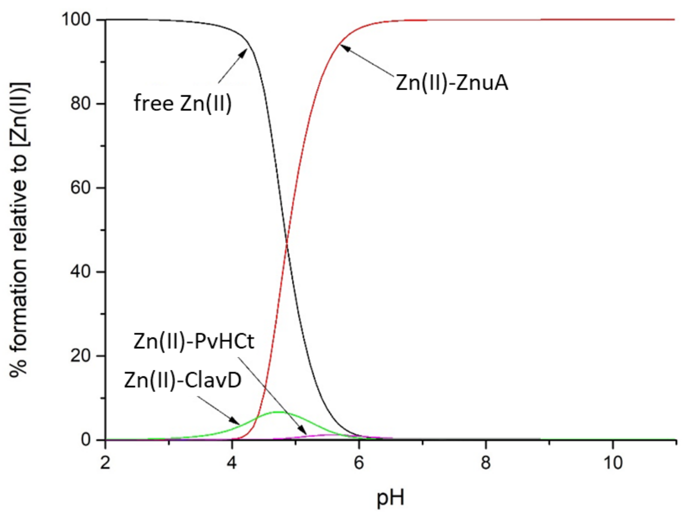

Fig. S3: A competition diagram between Zn(II) and PvHCt, clavanin D and the fragment of ZnuA transporter. Diagram presents a hypothetical situation when equimolar amounts of reagents are mixed. Clavanin D coordinates Zn(II) by three, and ZnuA fragment (Ac-$_{115}$MKSI-HGDDDDHDHAEKSDEDHHHGDFNMHLW145-NH_2), by four imidazole nitrogens. [Zn(II)] = [PvHCt] = [clavanin D] = [ZnuA] = 0.001 M.

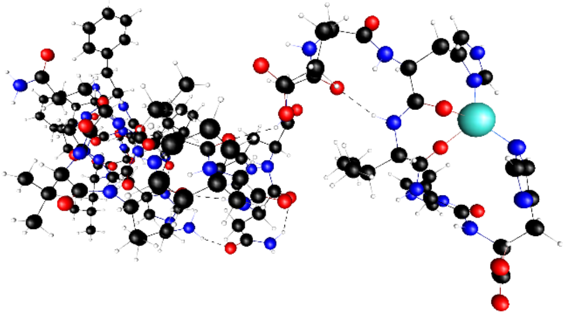

A

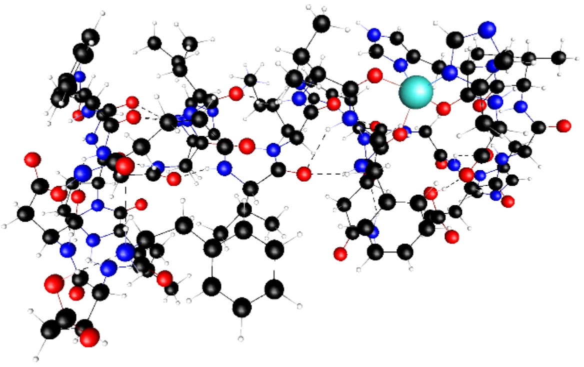

B

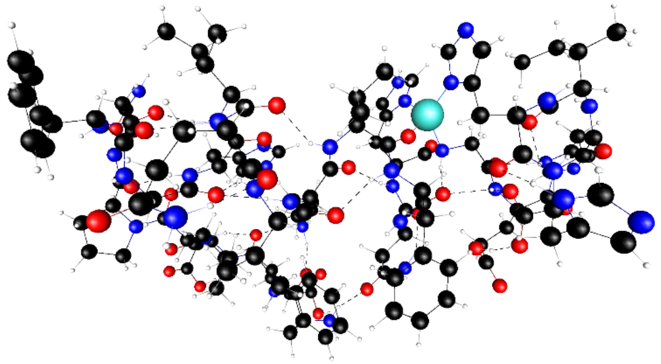

C

Fig. S4: The structures of three Zn(II)-PvHCt complexes. In (A), Zn(II) is bound to His20 and His23, in (B) – to His22 and His23, (C) – to His17 and His22.

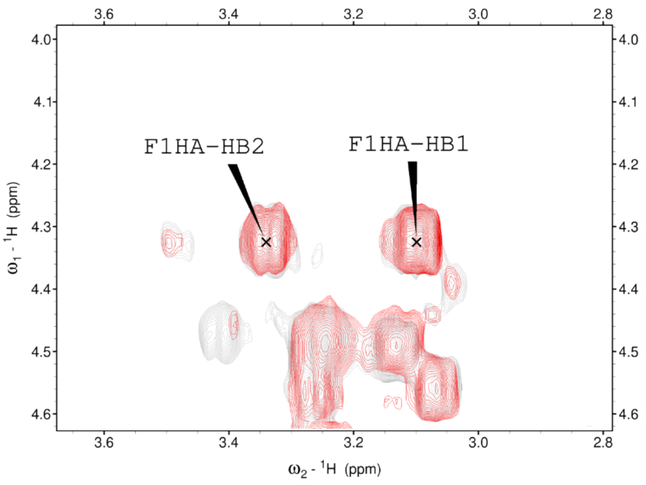

Fig. S5: Superimposition of aliphatic region of ^{1}H-^{1}H TOCSY spectra of PvHCt, 1 mM in absence (gray) and in presence of 0.2 Zn(II) eqs. in T=298 K (red), pH 7.4.

A)

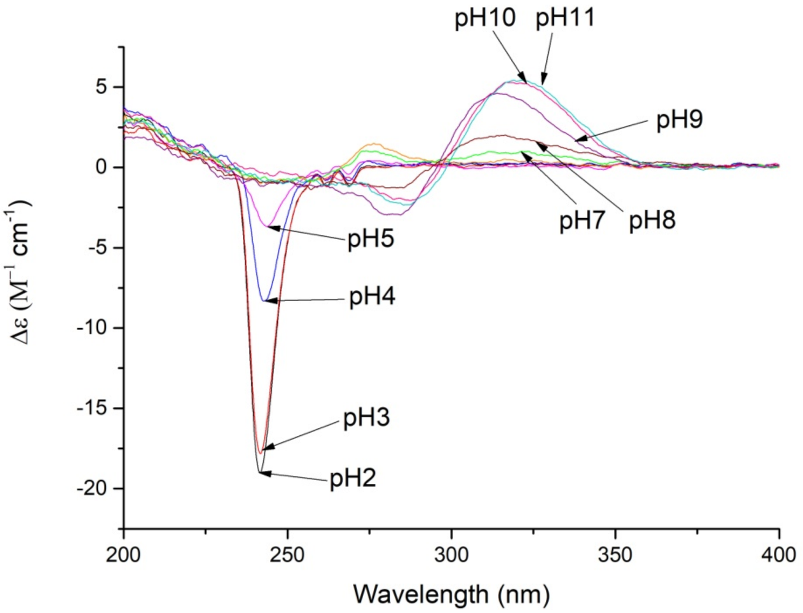

B)

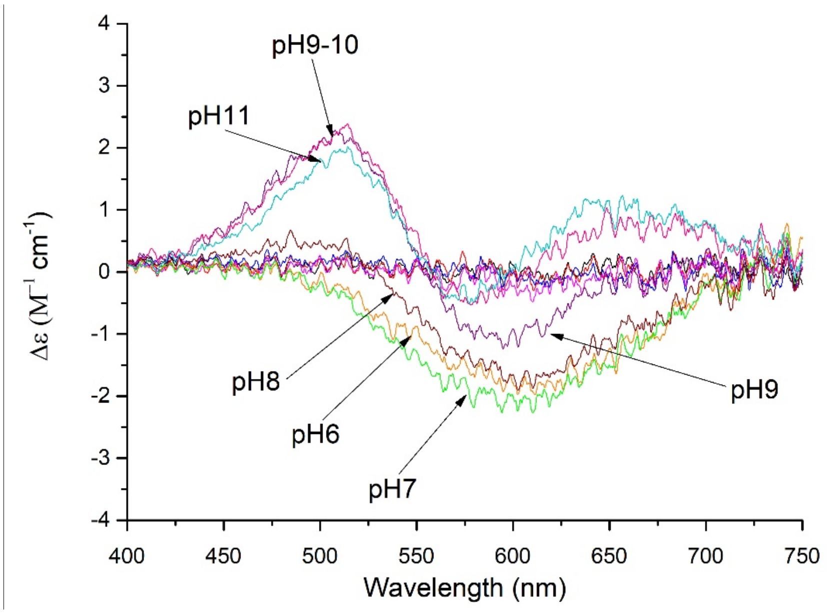

Fig. S6: Circular dichroism spectra of Cu(II) complexes with PvHCt in range A) 200-400 nm, B) 400-800 nm in pH range 2-11. Conditions: T = 298 K, *I* = 40 mM SDS, [Cu(II)] = [PvHCt] = 0.001 M.

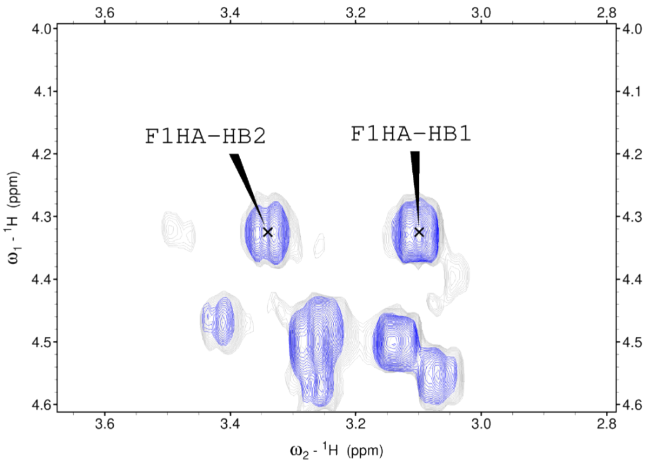

Fig. S7: Superimposition of aliphatic region of ^1H-^1H TOCSY spectra of PvHCt, 1 mM in absence (gray) and in presence of 0.1 Cu(II) eqs. in T=298 K (blue), pH 7.4.

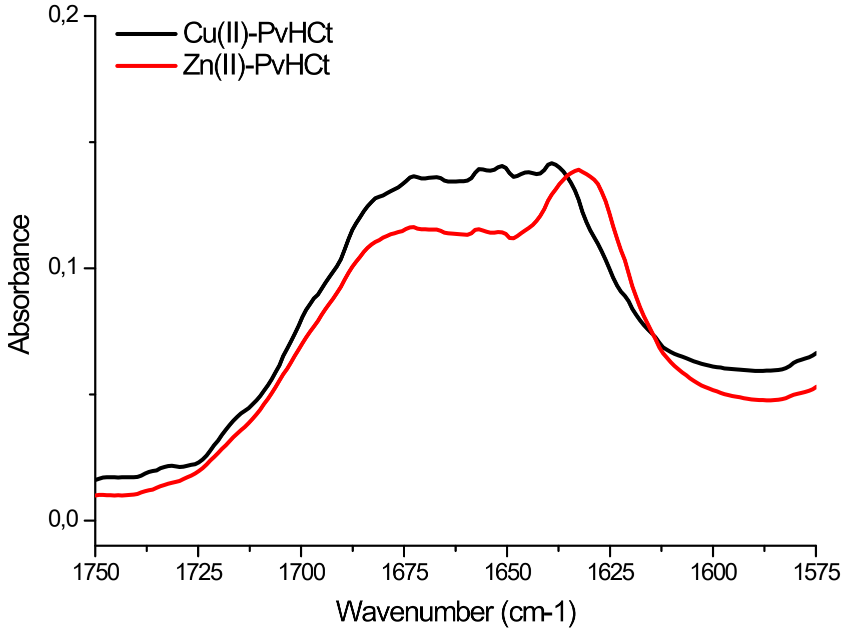

Fig. S8: FT-IR spectra of Cu(II) (black line) and Zn(II) bound (red line) forms of PvHCt. All the spectra were collected at T=298 K (blue), pH 7.4. The molar concentrations are 1.0 and 0.9 mM for the peptides and metal ions , respectively.

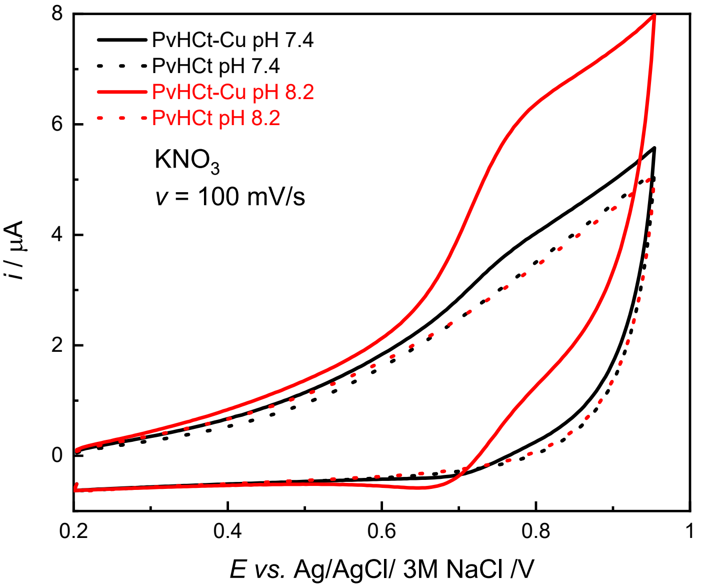

Fig. S9: CV obtained for Cu(II) complexes with PvHCt (1: 0.9 molar ratio) and PvHCt alone at pH 7.4 (black curves), 8.2 (red curves), recorded in 4 mM HNO_3/ 96 mM KNO_3 , v = 100 mV/s

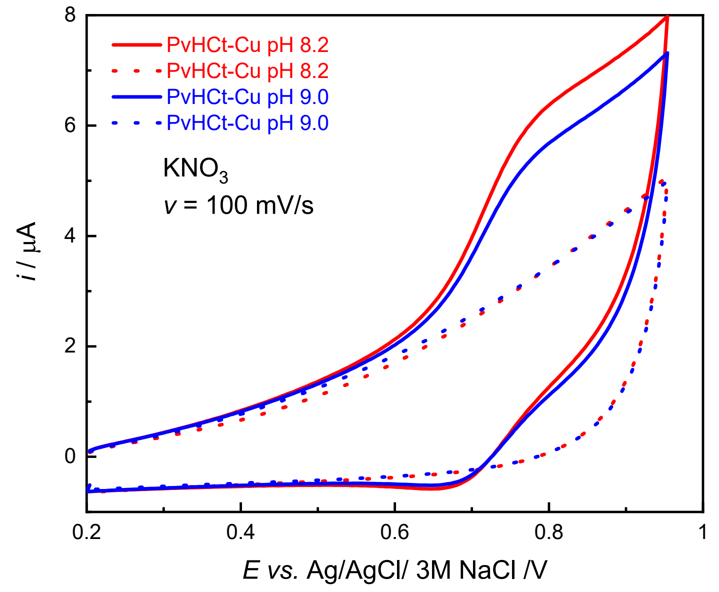

Fig. S10: CV obtained for Cu(II) complexes with PvHCt (1: 0.9 molar ratio) and PvHCt alone at 8.2 (red curves), 89,0 (blue curves), recorded in 4 mM HNO_3/ 96 mM KNO_3 , v = 100 mV/s

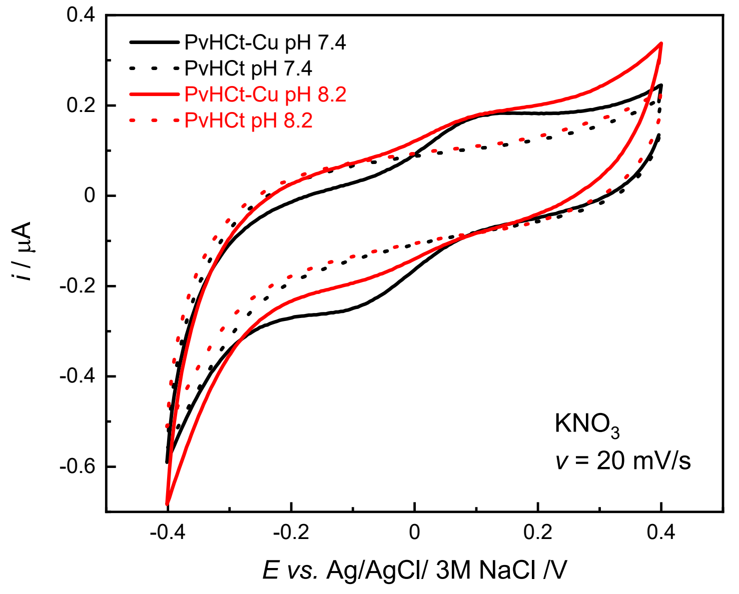

Fig. S11: CV obtained for Cu(II) complexes with PvHCt (1: 0.9 molar ratio) and PvHCt alone at pH 7.4 (black curves), 8.2 (red curves), recorded in 4 mM HNO_3/ 96 mM KNO_3 , v = 20 mV/s

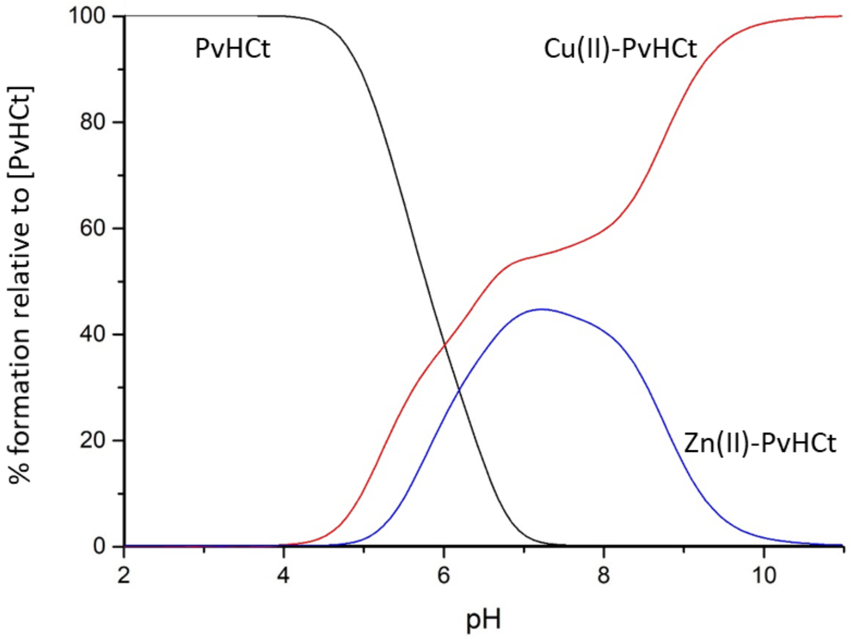

Fig. S12: A competition diagram between PvHCt, Zn(II) and Cu(II) ions. Diagram presents a hypothetical situation when equimolar amounts of reagents are mixed. It suggests that at pH around 7.4 the stability of complexes with Zn(II) and Cu(II) are comparable, with a slight advantage of the Cu(II) complex. [PvHCt] = [Zn(II)] = [Cu(II)] = 0.001 M.

References

1. Micsonai, A.; Moussong, É.; Wien, F.; Boros, E.; Vadászi, H.; Murvai, N.; Lee, Y.-H.; Molnár, T.; Réfrégiers, M.; Goto, Y.; Tantos, Á.; Kardos, J. BeStSel: webserver for secondary structure and fold prediction for protein CD spectroscopy. *Nucleic Acids Res* **2022,** *50* (W1), W90-W98.

2. Miles, A. J.; Ramalli, S. G.; Wallace, B. A. DichroWeb , a website for calculating protein secondary structure from circular dichroism spectroscopic data. *Protein Science* **2022,** *31* (1), 37–46.
